# Supplementary material for: Effect of Terpenoids and Flavonoids Isolated from Baccharis conferta Kunth on TPA-Induced Ear Edema in Mice
Source: Molecules. 2020 Mar 18;25(6):1379. doi: 10.3390/molecules25061379 (PMC7144369; doi:10.3390/molecules25061379)
Supplement: Supplementary file 1 [file molecules-25-01379-s001.pdf]

Supplementary Material

Effect of Terpenoids and Flavonoids Isolated from *Baccharis conferta* Kunth on TPA-Induced Ear Edema in Mice

Gutiérrez-Román Ana Silvia [1,2], Trejo-Tapia Gabriela [1]*, Herrera-Ruiz Maribel [2], Monterrosas-Brisson Nayeli [3], Trejo-Espino José Luis [1], Zamilpa Alejandro [2], and González-Cortazar Manasés [2]*

[1] Centro de Desarrollo de Productos Bióticos. Instituto Politécnico Nacional (IPN), Col. San Isidro, Carretera Yautepec-Jojutla, Km 6, 62731, Morelos, México. E-Mail: gttapia@ipn.mx (G.T.-T.); 12gtr.ana@gmail.com (A.G.-R.); jtrejo@ipn.mx (J.T.-E)

[2] Centro de Investigación Biomédica del Sur, Instituto Mexicano del Seguro Social, Argentina 1, Col. Centro, Xochitepec, 62790, Morelos, México. E-Mails: gmanases@hotmail.com (M.G.-C.); cibis_herj@yahoo.com.mx (M.H-R.), azamilpa_2000@yahoo.com.mx (A.Z.).

[3] Facultad de Ciencias Biológicas, Universidad Autónoma del Estado de Morelos (UAEM), Av. Universidad 1001, Col. Chamilpa, Cuernavaca, 62209, Morelos, México. E-Mails: nmonterrosas78@gmail.com (N.M.-B.)

* Correspondence: gmanases2000@gmail.com (Manasés González-Cortazar); Tel.: +52 (777) 3612155; gttapia@ipn.com (Gabriela Trejo-Tapia); Tel: +52 (735) 3942020

Table.S1. ^{1}H and ^{13}C NMR spectroscopy data of schensianol A (**1**, $CDCl_3$).

Position	$\delta^{1}H$ (*J* in Hz)	$\delta^{13}C$
1a	5.06 (d, 11)	111.1
1b	5.20 (d, 17.2)	-
2	-	144.8
3	-	72.6
4a	2.29 (d, 7.7)	45.1
4b	2.24 (d, 7.7)	-
5	-	122.1
6	-	140.3
7	-	82.4
8	-	38.1
9	-	26.6
10	-	85.6
11	-	71.1
12	1.12 s	24.4
13	1.24 s	26.6
14	1.32 s	27.5
15	1.27 s	27.2

Table.S2. ^{1}H and ^{13}C NMR spectroscopy data of cirsimaritin and hispidulin (**6** and **7**, CD_3OD).

Position	δ ^{1}H (*J* in Hz)	$\delta^{13}C$	δ ^{1}H (*J* in Hz)	$\delta^{13}C$
	**6**	**6**	**7**	**7**
2	--	164.1	--	165.4
3	6.37, s	102.7	6.38, s	102.0
4	--	182.0	--	183.0
5	--	152.6	--	153.2
6	--	131.8	--	136.1
7	--	158.0	--	156.3
8	6.40, s	90.1	6.26, s	98.4
9	--	152.3	--	152.5
10	--	105.3	--	101.9
1′	--	121.2	--	122.2
2′	7.58, d, (8.1)	127.5	7.74, d, (8.8)	129.1
3′	6.78, d, (8.1)	115.7	6.84, d, (8.8)	118.0
4′	--	160.8	--	165.4
5′	6.78, d, (8.1)	115.7	6.84, d, (8.8)	118.0
6′	7.58, d, (8.1)	127.5	7.74, d, (8.8)	129.1
4-OCH_3	3.73, s	55.9	--	--
6-OCH_3	3.91, s	60.3	3.82, s	60.5

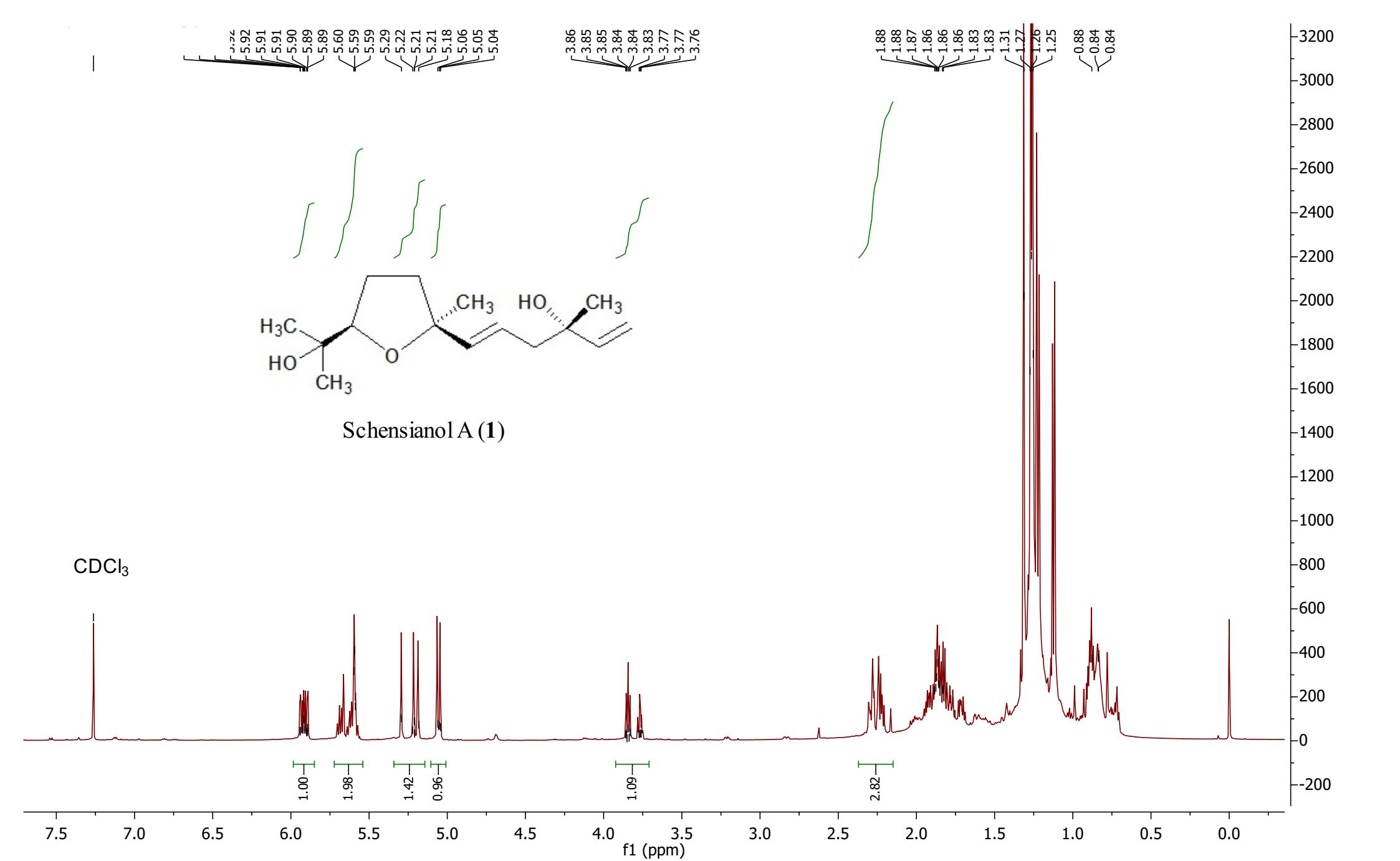

Fig. S1. ^{1}H-NMR ($CDCl_3$, 600 MHz) compound (**1**)

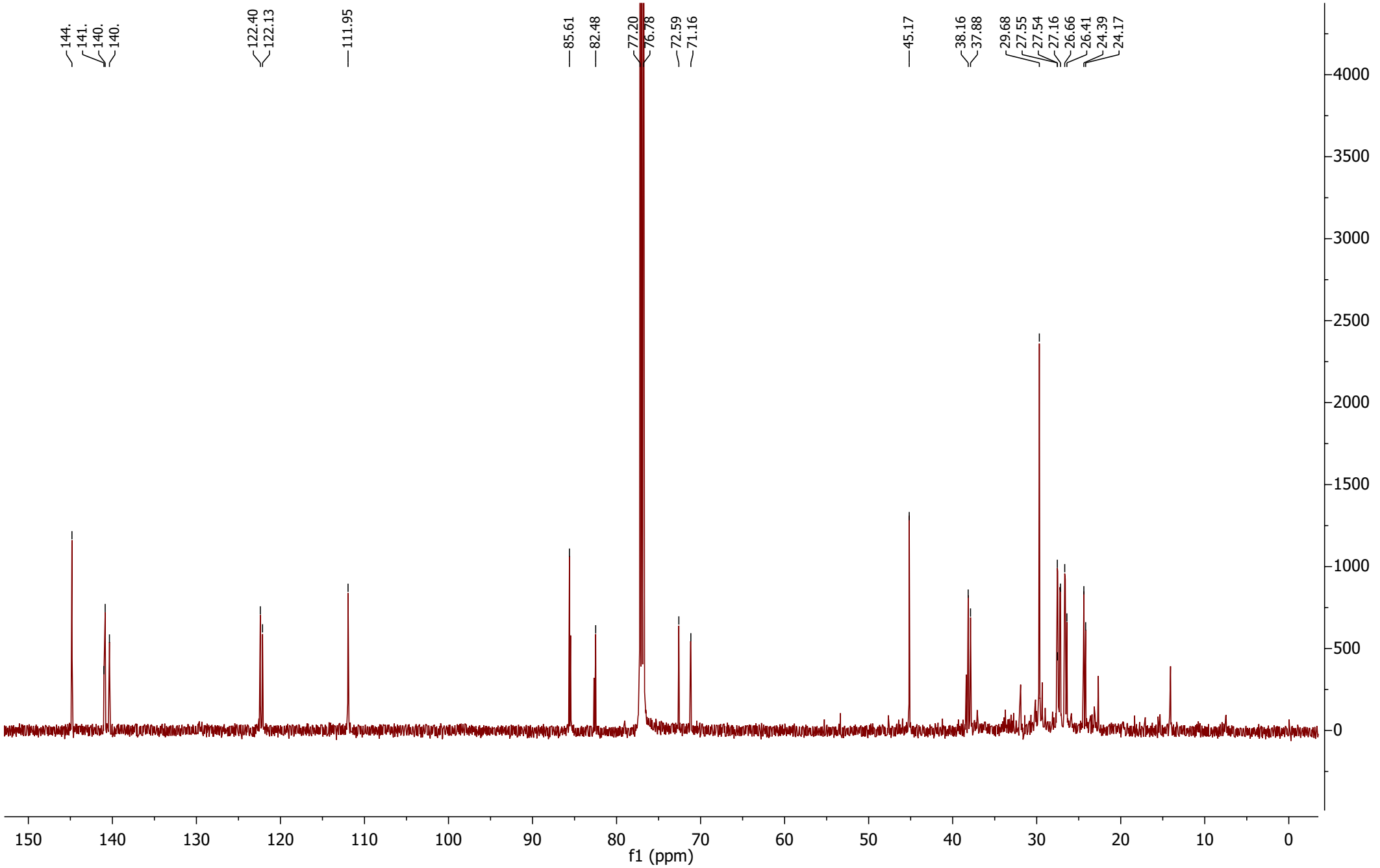

Fig. S2. ^{13}C-NMR ($CDCl_3$, 600 MHz) compound (**1**)

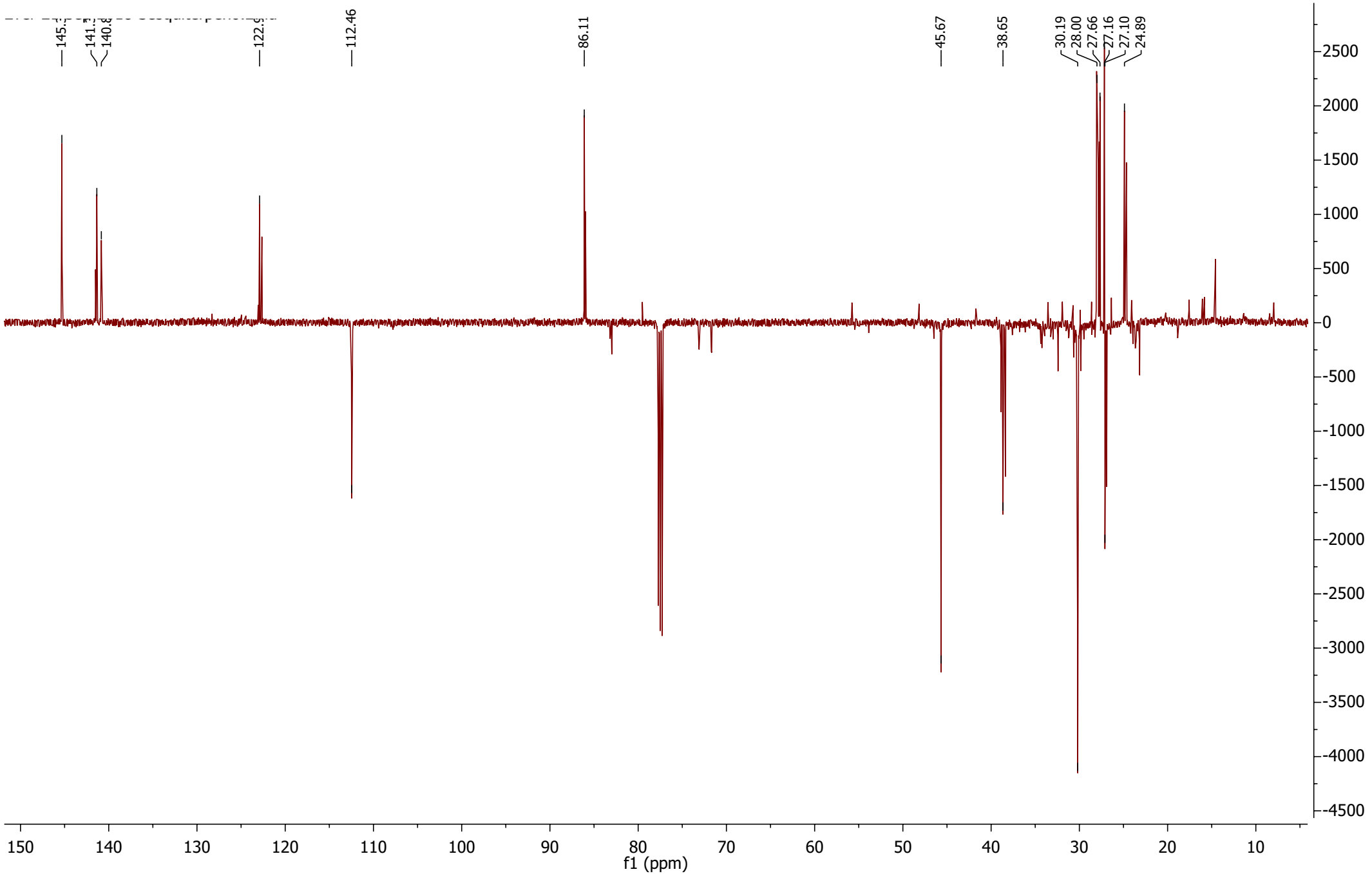

Fig. S3. ^{13}C(DEPT)-NMR ($CDCl_3$, 600 MHz) compound (**1**)

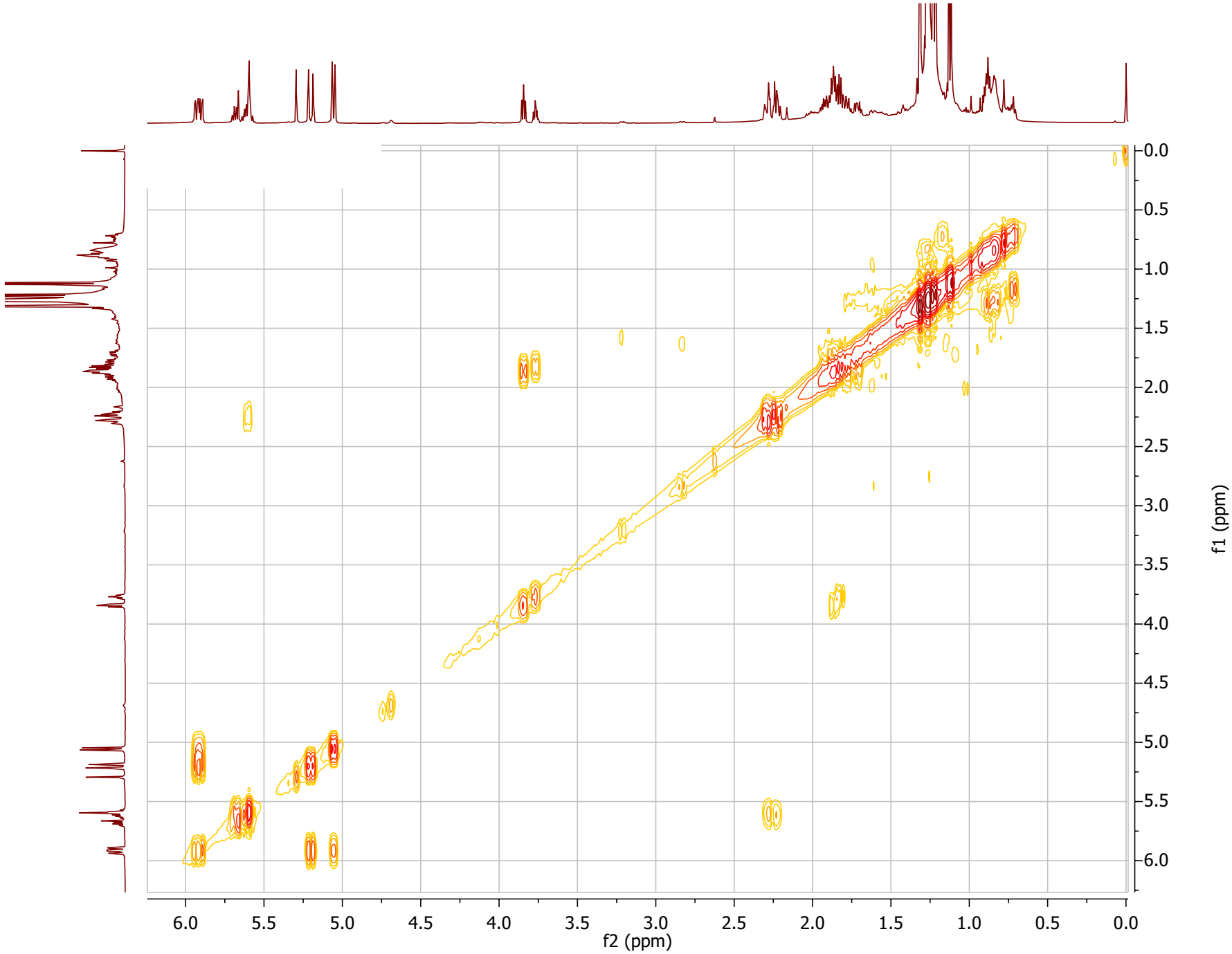

Fig. S4. ^{1}H-^{1}H(COSY)-NMR ($CDCl_3$, 600 MHz) compound (**1**)

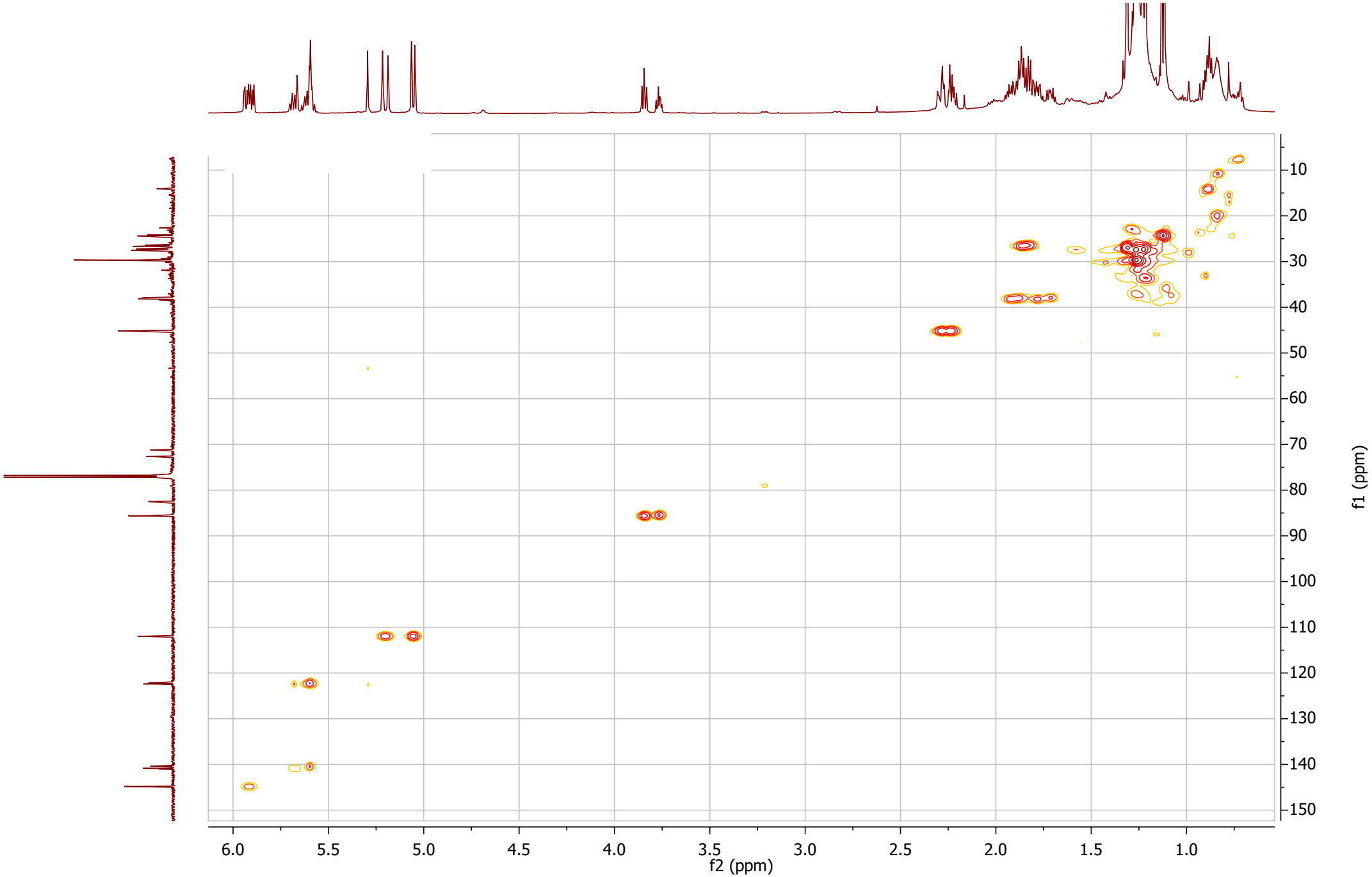

Fig. S5. ^{1}H-^{13}C(HSQC)-NMR ($CDCl_3$, 600 MHz) compound (**1**)

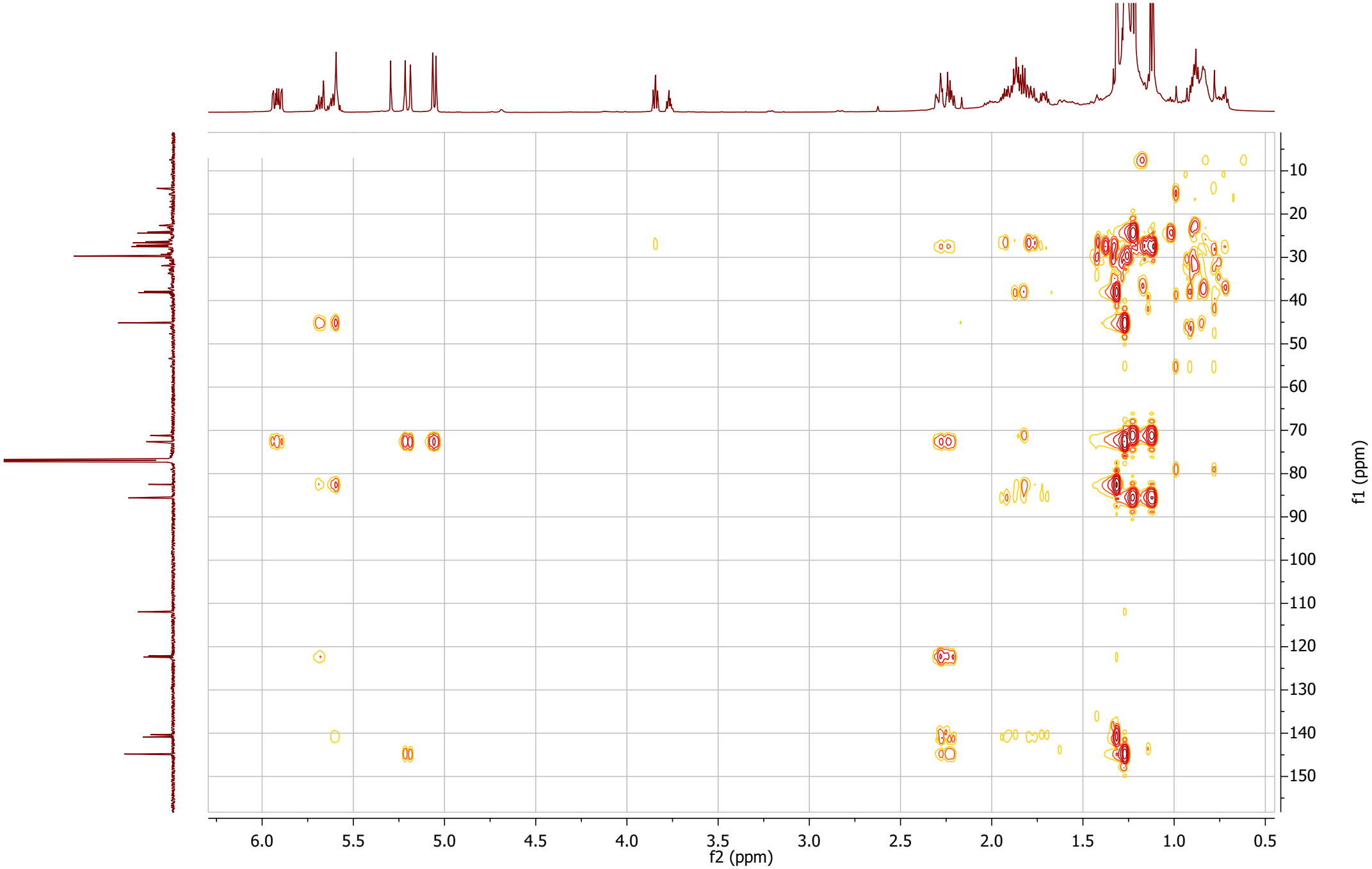

Fig. S6. ^{1}H-^{13}C(HMBC)-NMR ($CDCl_3$, 600 MHz) compound (**1**)

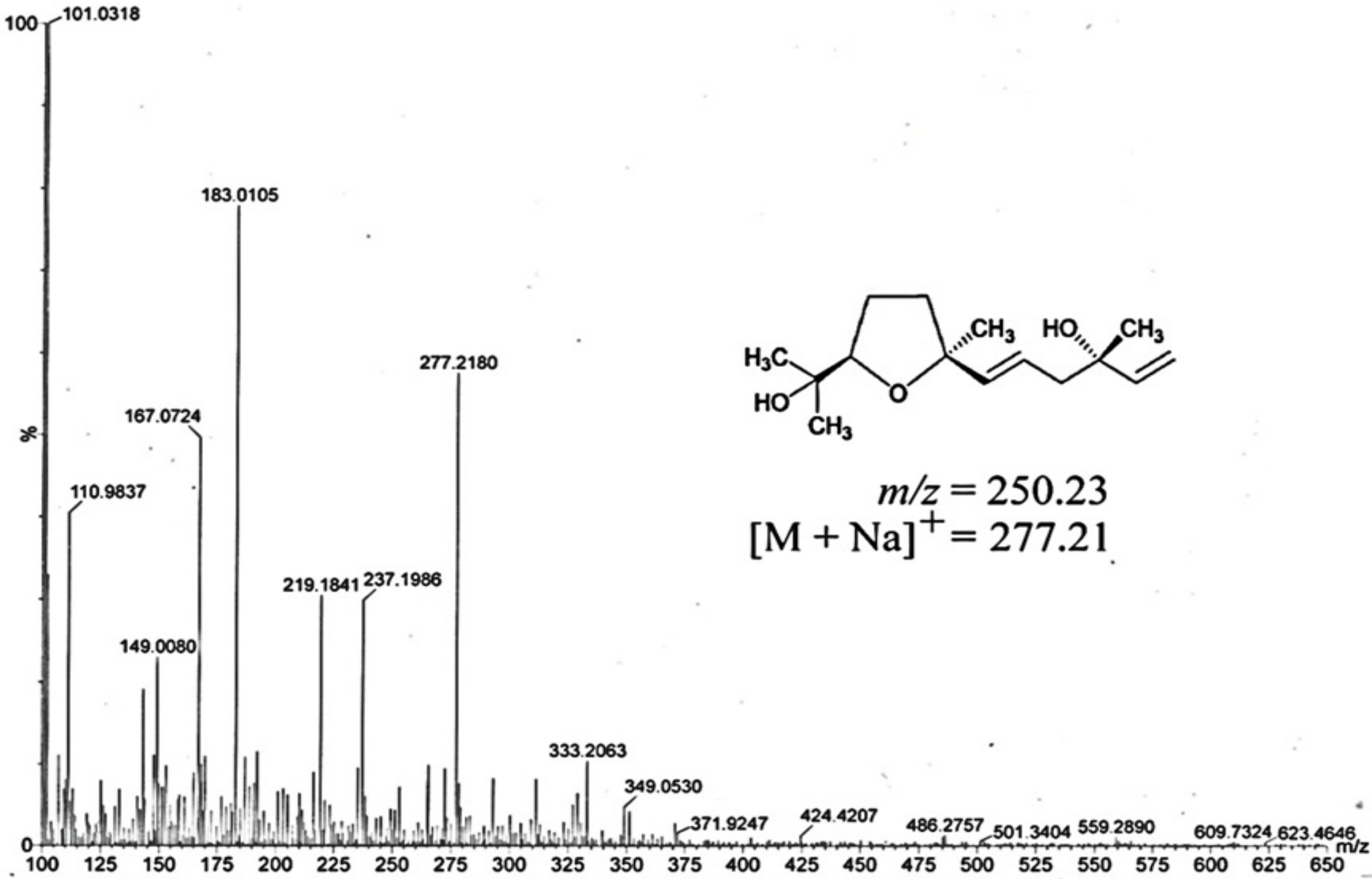

Fig. S7. EI-MS spectrum compound (**1**)

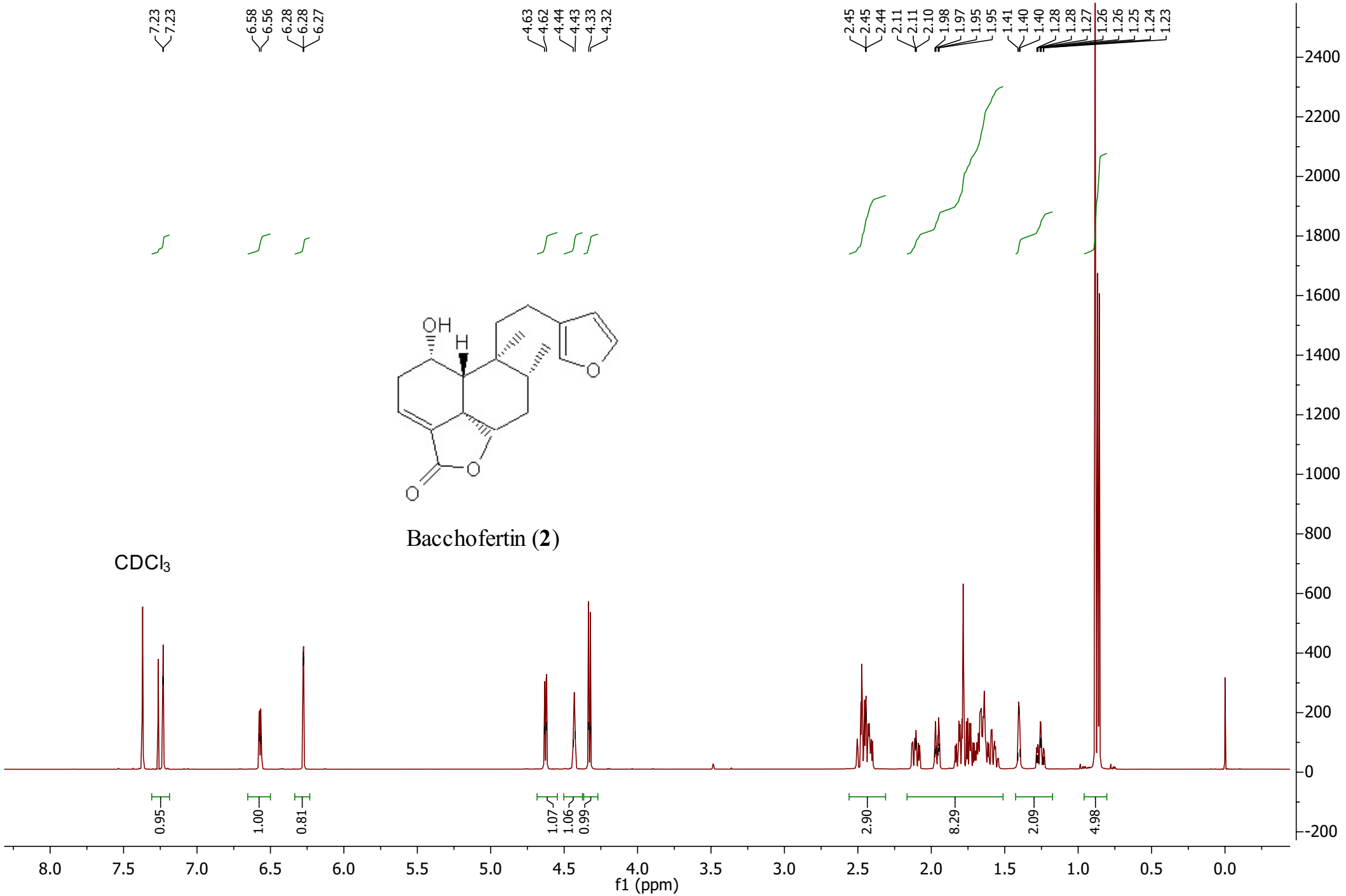

Fig. S8. ^{1}H-NMR ($CDCl_3$, 600 MHz) compound (**2**)

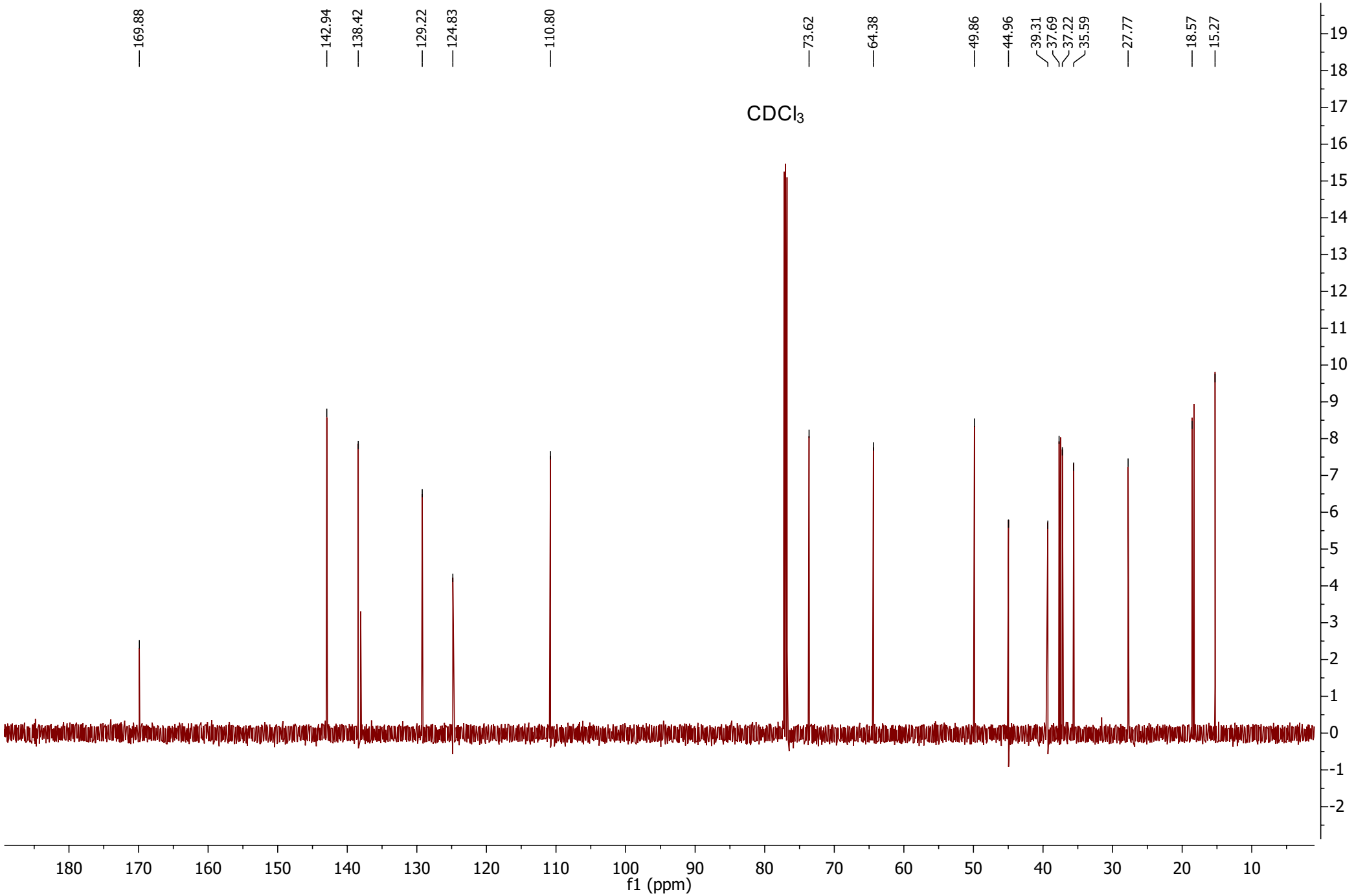

Fig. S9. ^{13}C-NMR ($CDCl_3$, 600 MHz) compound (**2**)

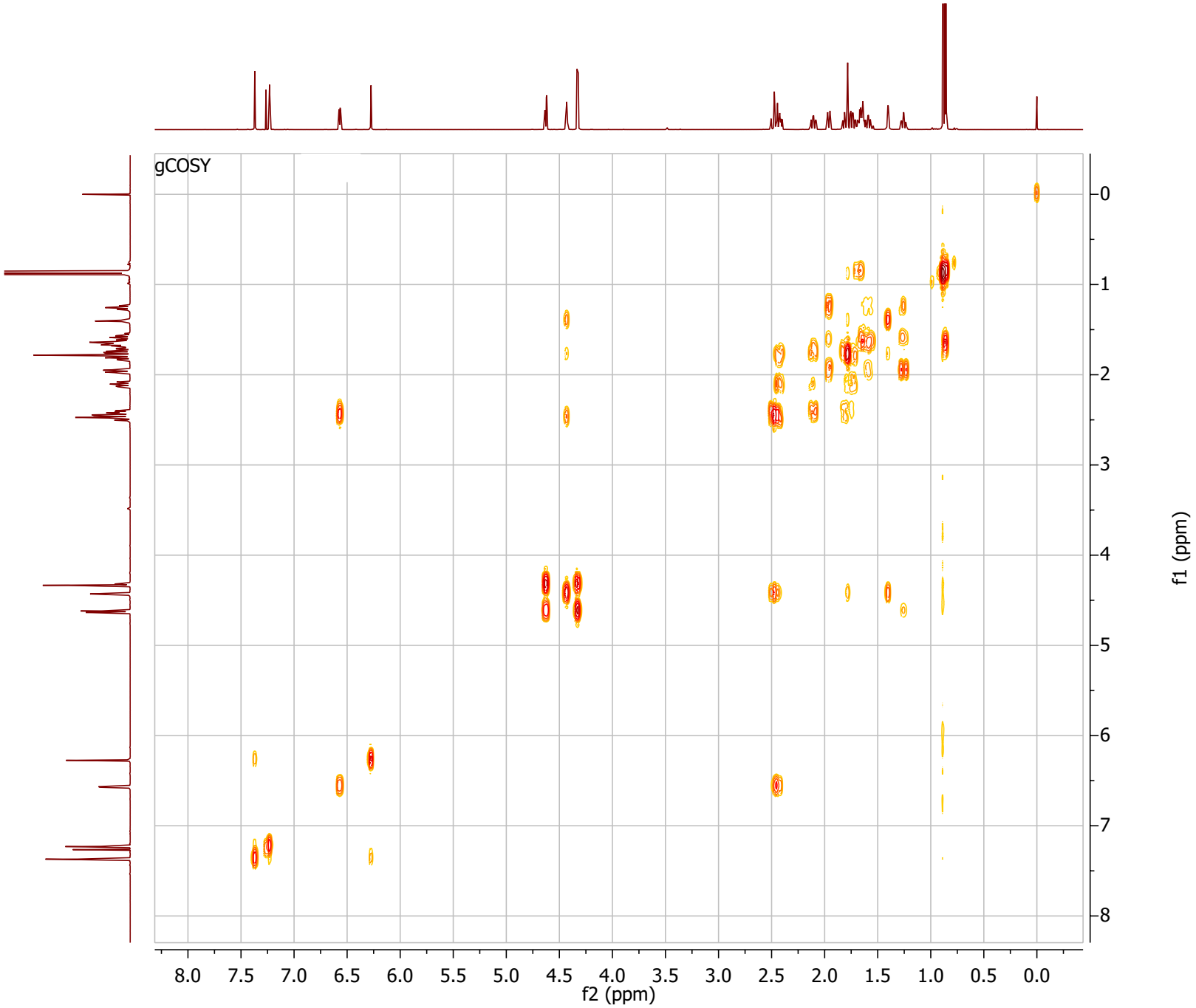

Fig. S10. ^{1}H-^{1}H(COSY)-NMR ($CDCl_3$, 600 MHz) compound (**2**)

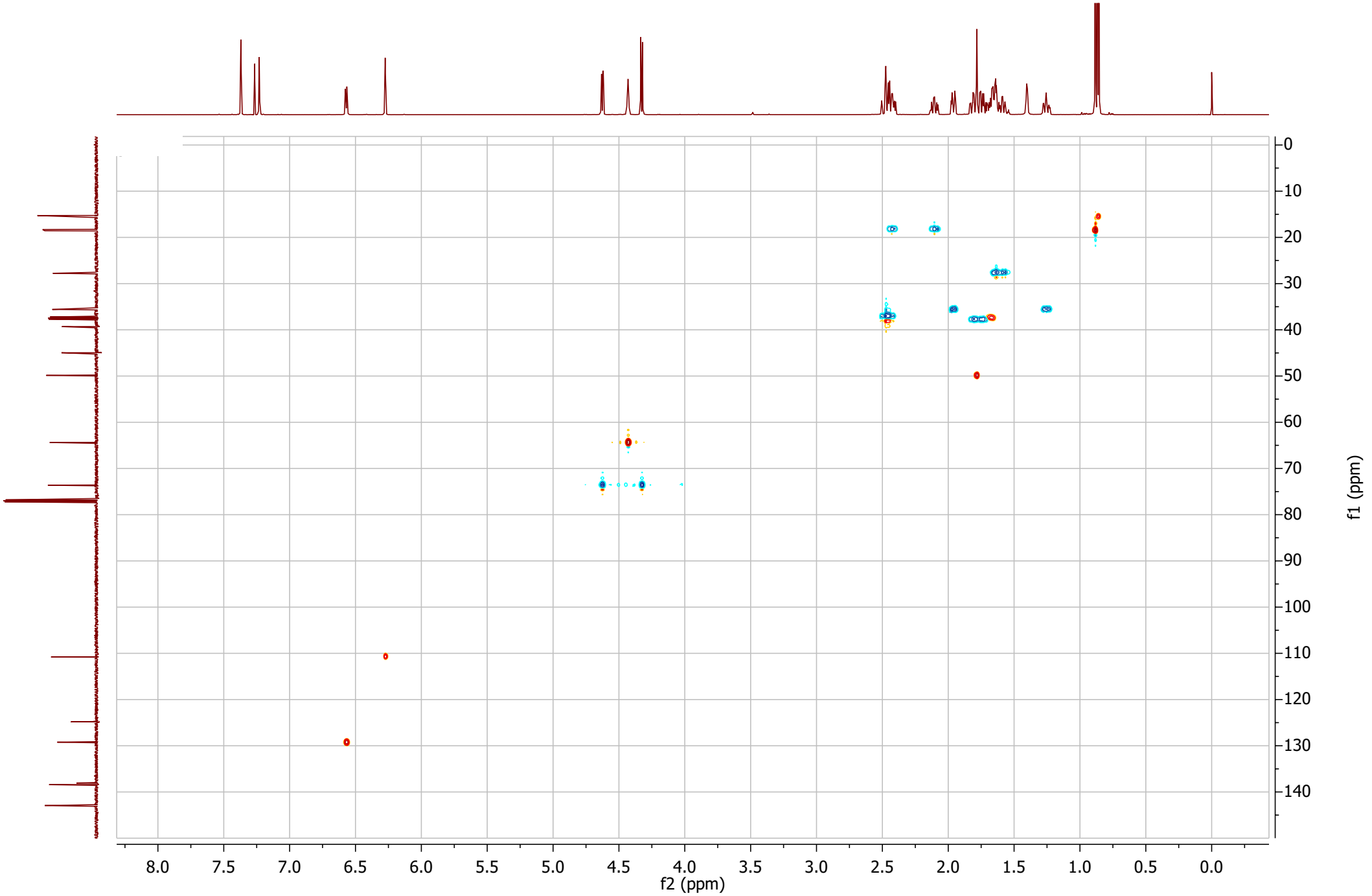

Fig. 11. ^{1}H-^{13}C(HSQC)-NMR ($CDCl_3$, 600 MHz) compound (**2**)

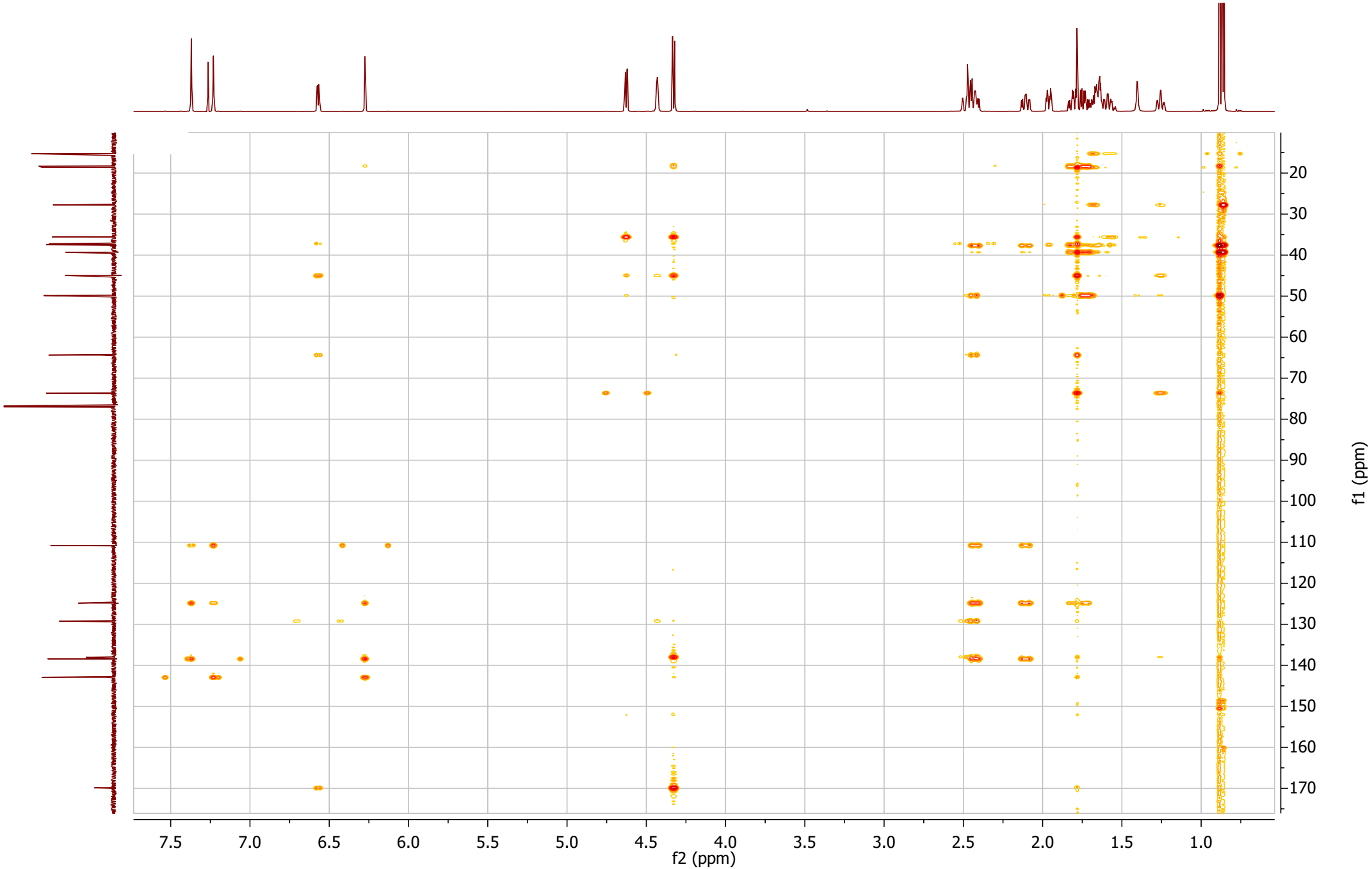

Fig. S12. ^{1}H-^{13}C(HMBC)-NMR ($CDCl_3$, 600 MHz) compound (**2**)

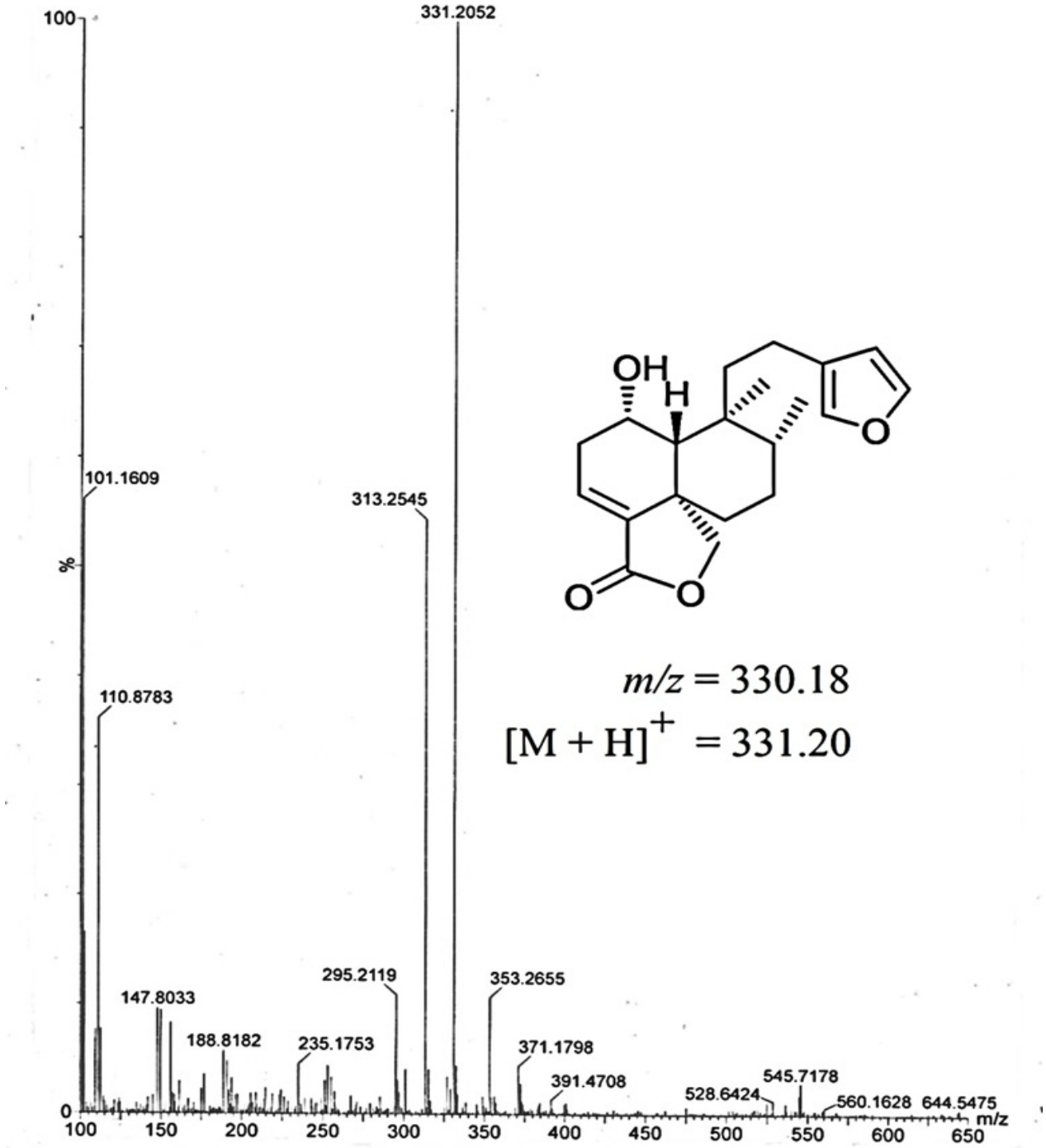

Fig. S13. EI-MS spectrum compound (**2**)

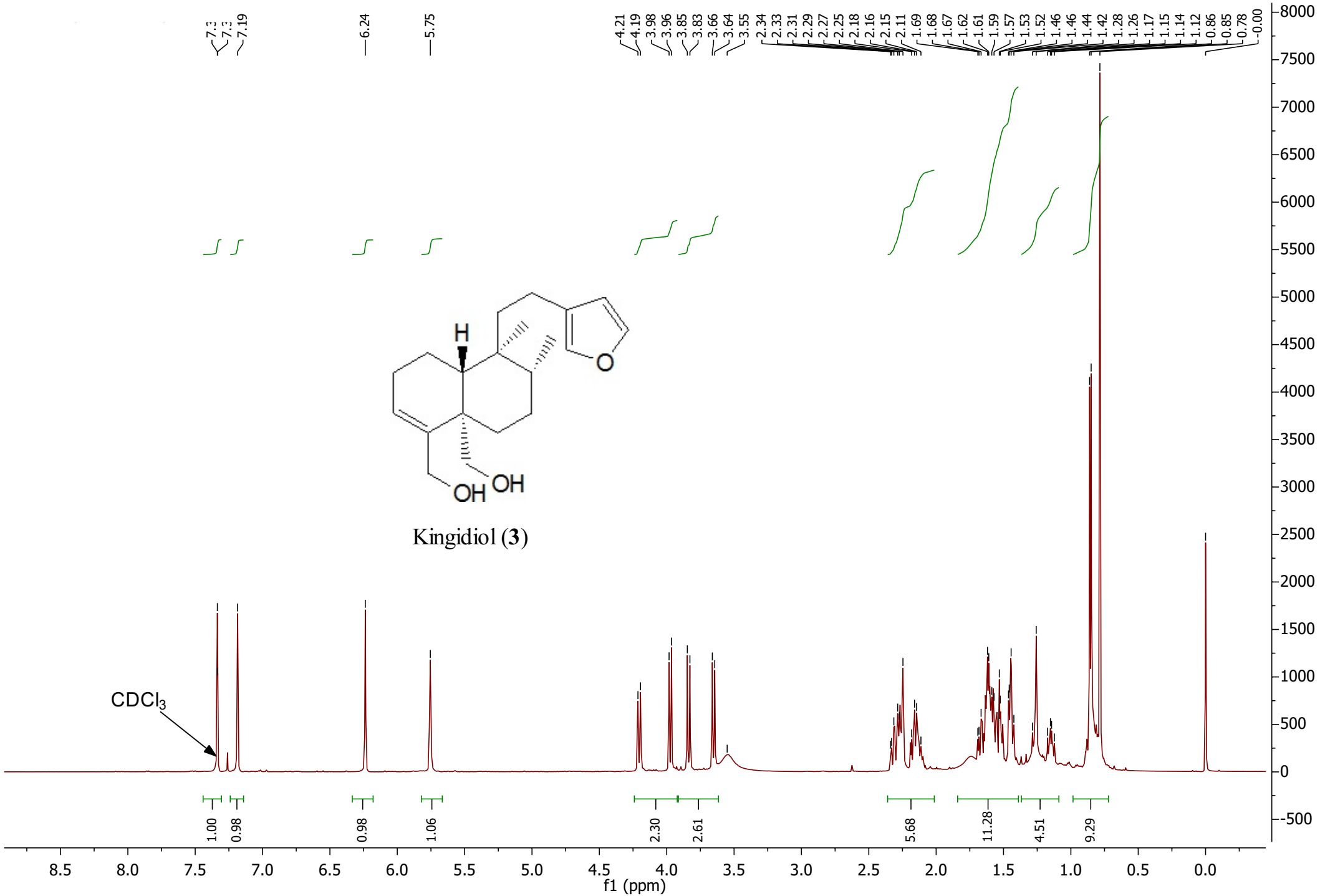

Kingidiol (**3**)

Fig. S14. ^{1}H-NMR ($CDCl_3$, 600 MHz) compound (**3**)

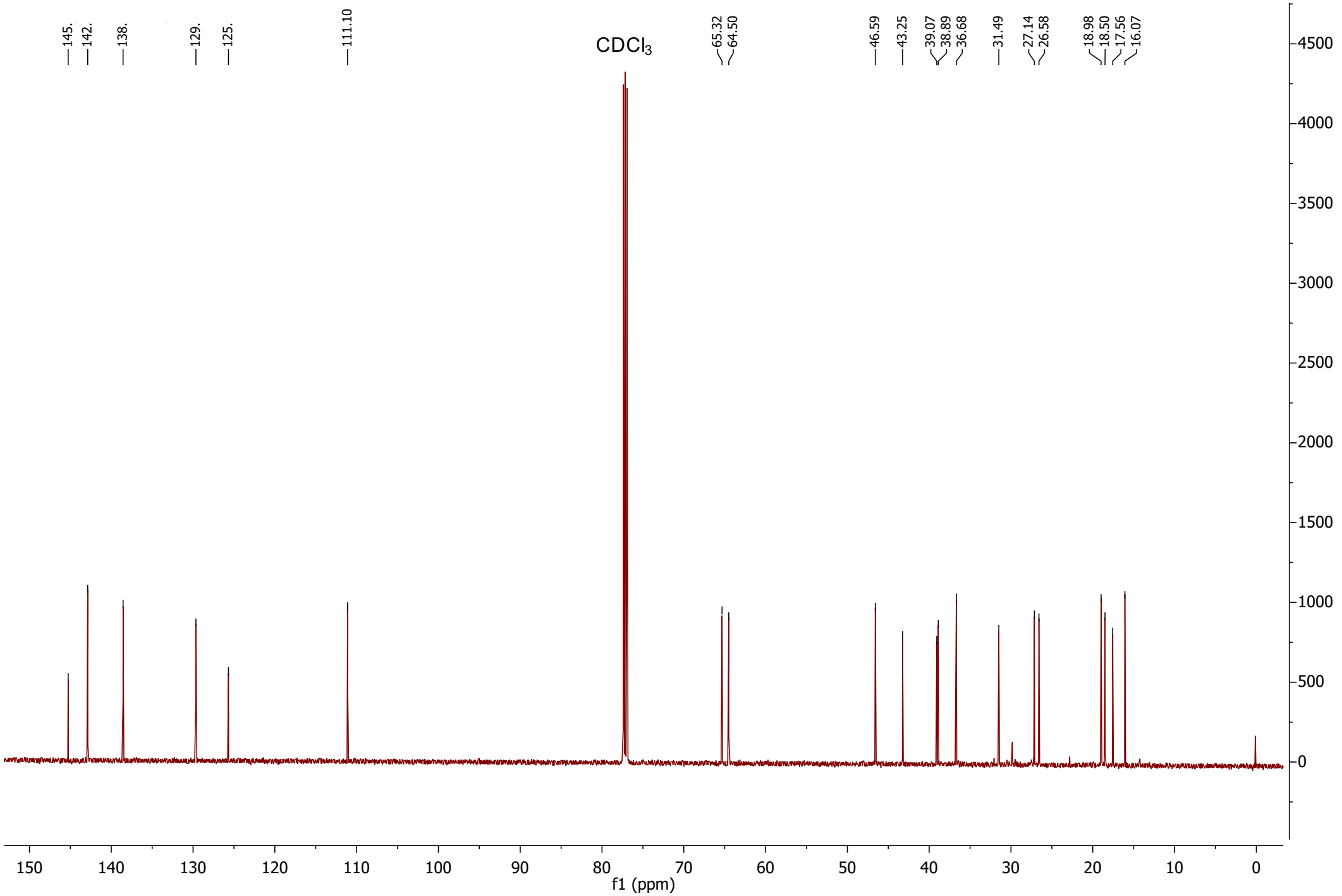

Fig. S15. ^{13}C-NMR ($CDCl_3$, 600 MHz) compound (**3**)

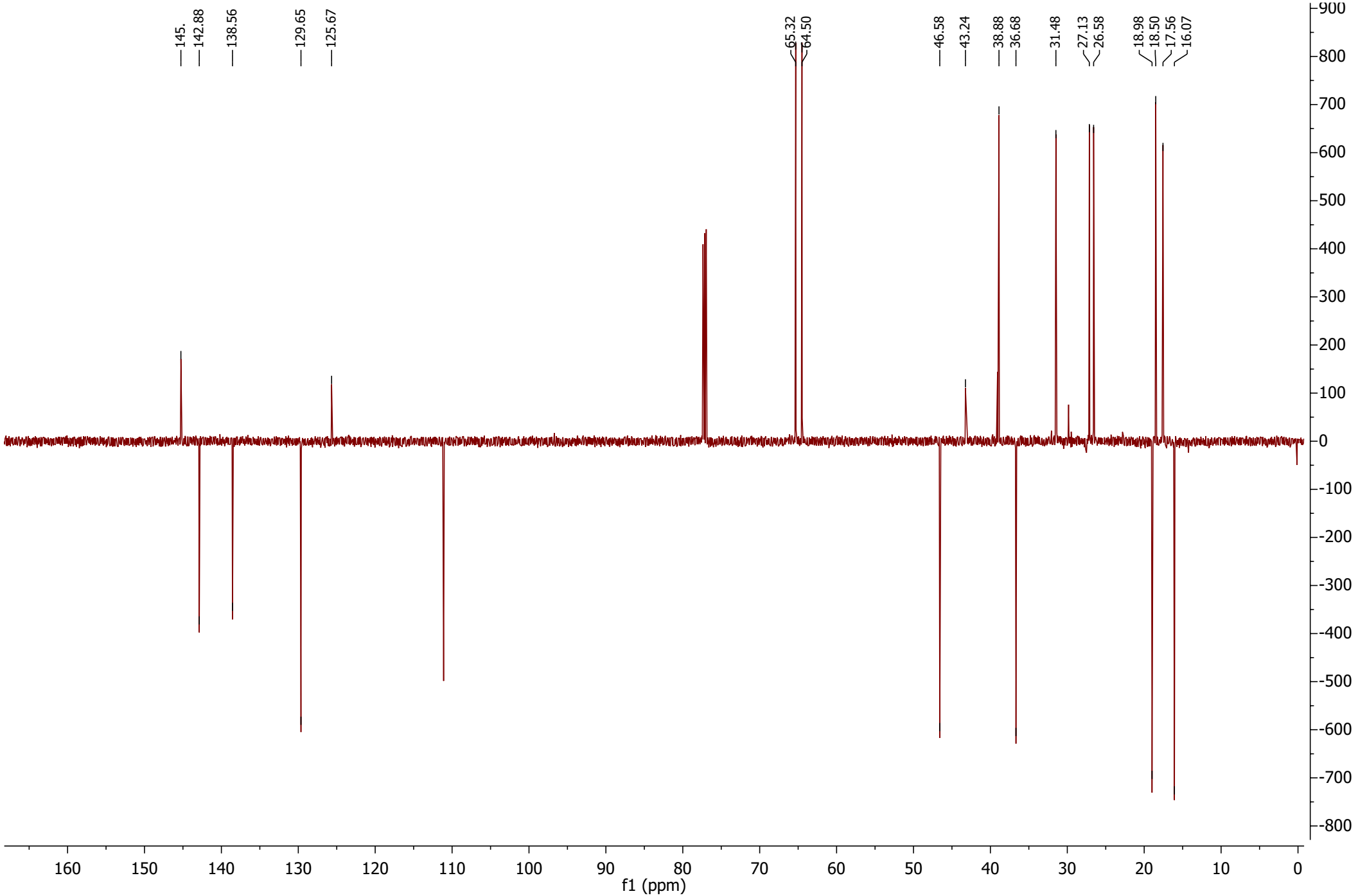

Fig. S16. ^{13}C(DEPT)-NMR ($CDCl_3$, 600 MHz) compound (**3**)

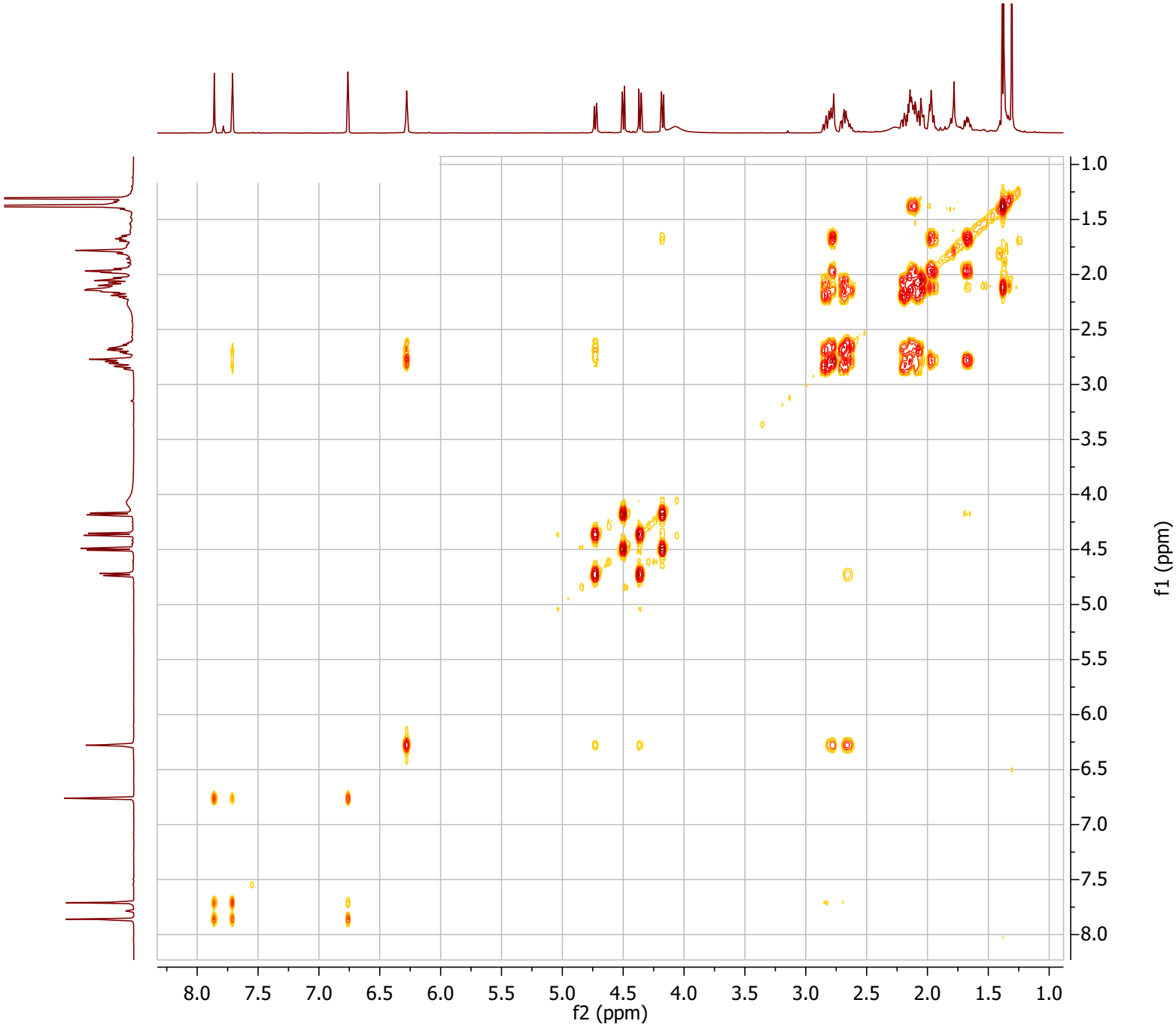

Fig. S17. ^{1}H-^{1}H(COSY)-NMR ($CDCl_3$, 600 MHz) compound (**3**)

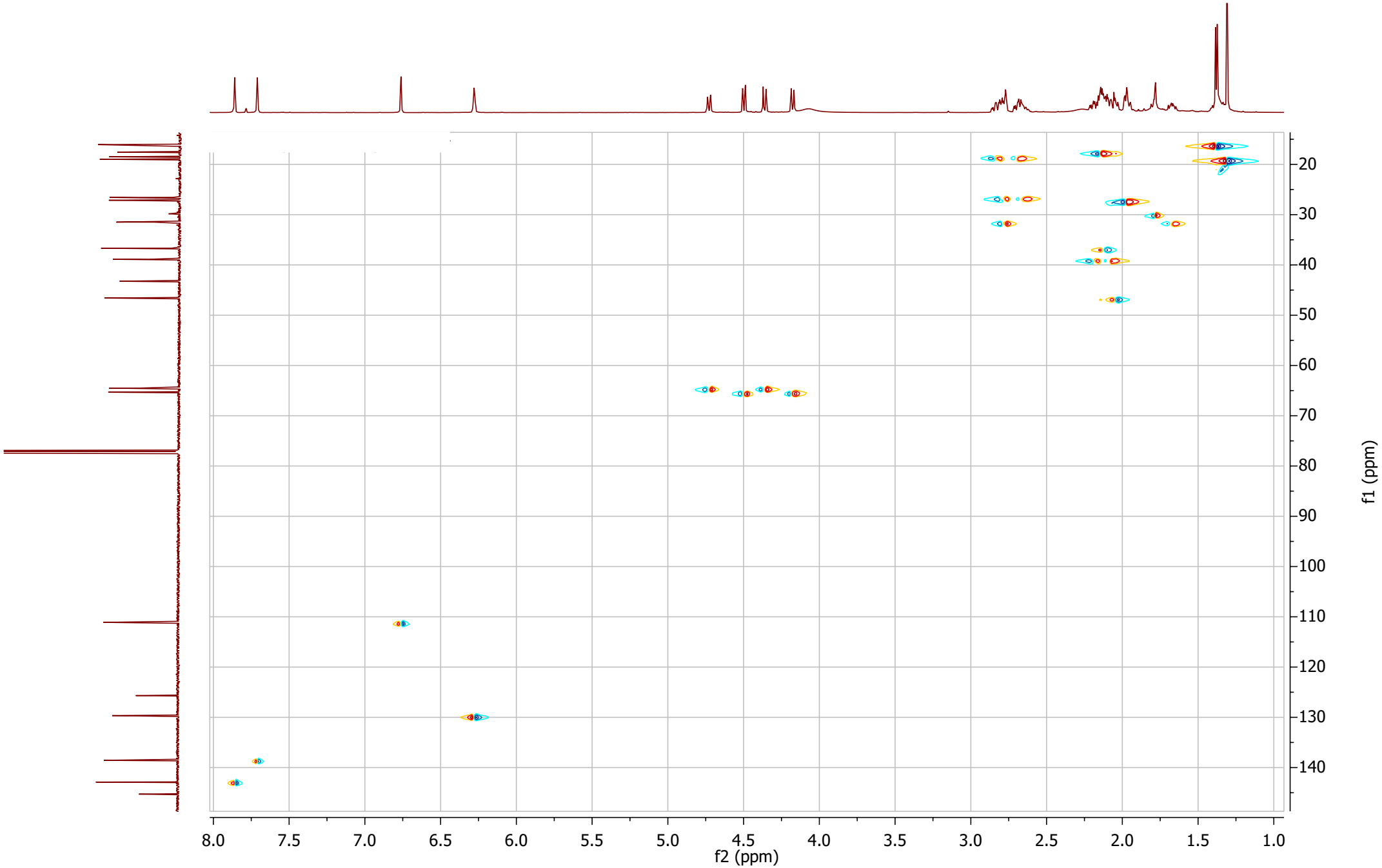

Fig. S18. ^{1}H-^{13}C(HSQC)-NMR ($CDCl_3$, 600 MHz) compound (**3**)

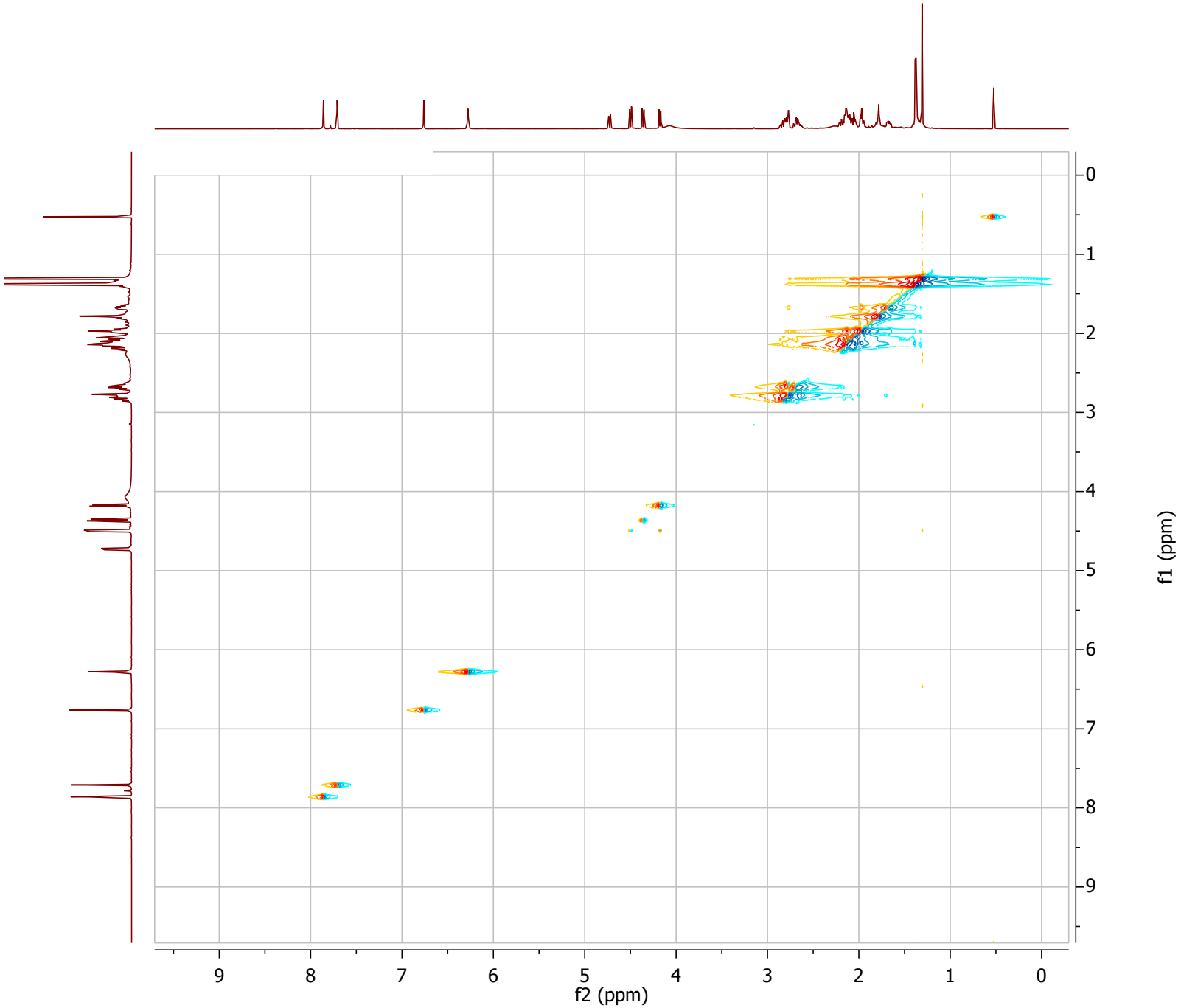

Fig. S19. ^{1}H-^{1}H(NOESY)-NMR ($CDCl_3$, 600 MHz) compound (**3**)

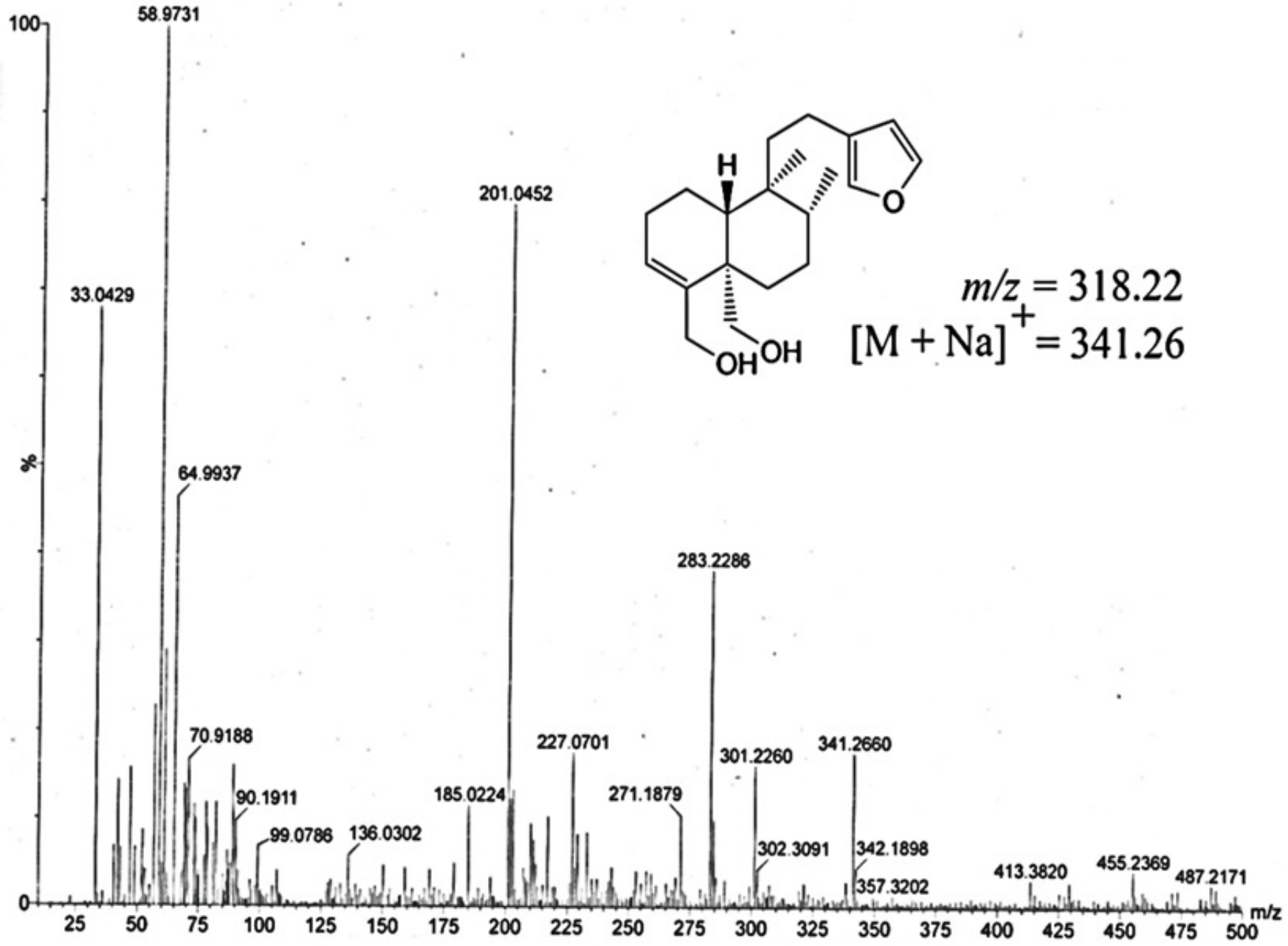

Fig. S20. EI-MS spectrum compound (**3**)
